# Are CSF Pressure Factors Related to the Development of Post-dural Puncture Headache?

**DOI:** 10.3389/fneur.2019.00700

**Published:** 2019-07-02

**Authors:** Seong Hoon Kim, Tae Won Kim, Hae Eun Shin, Si Baek Lee, Dong Woo Ryu, Jeong Wook Park

**Affiliations:** ^1^Department of Neurology, College of Medicine, Uijeongbu St. Mary's Hospital, The Catholic University of Korea, Uijeongbu, South Korea; ^2^Department of Neurology, College of Medicine, Incheon St. Mary's Hospital, The Catholic University of Korea, Incheon, South Korea

**Keywords:** post dural puncture headache, CSF opening pressure, CSF closing pressure, cerebrospinal elastance, pressure volume index

## Abstract

Post-dural puncture headache (PDPH) is an unfavorable situation seen in considerable number of patients even though atraumatic and small needle reduces its incidence. CSF pressures measured at the time of puncture change after CSF drainage. In the present study, we investigated relationships between CSF pressure-related factors and occurrence of PDPH. We prospectively enrolled 103 participants who underwent CSF studies for meningitis. Using a standardized protocol, CSF opening pressure (OP) and closing pressure (CP) were measured, and cerebrospinal elastance (ECS) and pressure-volume index (PVI) were investigated. Within 14 days after dural puncture, we confirmed PDPH. According to PDPH development, the CSF pressure factors and clinical variables were compared between PDPH and non-PDPH group. Of the 103 participants, 100 (97.0%) had decreased CP, 16 (15.5%) had values below 6 cmH_2_O and the pressure change after dural puncture (OP-CP) was 6.1 ± 3.1 cmH_2_O. PVI and ECS measured by CSF drainage were 99.8 ± 89.5 and 0.4 ± 0.2 cmH_2_O/mL. Among the demographic factors, body weight was correlated with OP (*r* = 0.27), CP (*r* = 0.35), and PVI (*r* = 0.20). Height was weakly correlated with CP (*r* = 0.199) During the study period, 22 participants (21.34%) developed PDPH. None of the CSF pressure factors were significantly different between the PDPH and non-PDPH group and did not contributed to the development of PDPH. CSF pressure factors might not be related to the development of PDPH.

## Introduction

Dural puncture is essential for the diagnosis of CNS infections and disorders related to CSF dynamics (1). Post-dural puncture headache (PDPH) occurs in a considerable number of patients and causes difficulties for clinicians. Previous studies reported that the overall prevalence of PDPH was 20–40% with a conventional method, but smaller size and atraumatic needle could reduce the incidence to 3~6% (1, 2). In most cases, PDPH occurs within 24–48 h after dural puncture, and ICHD-3 is defined as a headache within 5 days after CSF drainage (3). PDPH is usually accompanied by neck stiffness and/or subjective hearing symptoms. Even though the majority of patients recover spontaneously within 2 weeks, some require treatment with autologous epidural lumbar patches.

Several hypotheses have been presented explaining the pathogenesis of PDPH, such as traction of pain-sensitive structures such as the meninges or cranial nerve occurring when upright and vasodilation to maintain intracranial volume according to the Monro-Kellie doctrine (4, 5). Although 7.2.1 PDPH is classified as a subcategory of 7.2, the headache attributed to low CSF pressure in ICHD-3 and low CSF pressure or dry-tapping, which is frequently seen clinically, studies of the role of CSF pressure as a mechanism underlying the development of PDPH are still inconclusive (6–8). In clinical practice, it is often difficult to repeat dural punctures in order to measure CSF pressure in patients with suspected PDPH.

Along with opening pressure (OP), pressure measured immediately after CSF drainage is referred to as closing pressure (CP). It is plausible to hypothesize that CP, more than OP, reflects the CSF pressure environment at the time of PDPH generation. Measurements of CP might be helpful in situations of idiopathic intracranial hypertension (IIH) and hydrocephalus, because the primary purpose of management is reducing pressure through CSF drainage (9, 10). In IIH, recurrent dural puncture and checking of CSF pressure have low therapeutic value, but pressure changes associated with CSF drainage provide helpful information for the prognosis of EVD and CSF shunts (9, 11).

The change in pressure between OP and CP (OP-CP) roughly reflects volume of the CSF reservoir (12). Craniospinal elastance (ECS), which is the change of pressure per change in removed CSF volume (ΔP/ΔV), and the pressure-volume index (PVI), which is the calculated volume required to raise CSF pressure by a factor of 10, have been used as parameters to predict compliance of the CSF space (13, 14). ECS and PVI have been investigated in patients with spontaneous CSF leakage and idiopathic intracranial hypertension (13, 15).

In the present study, we investigated whether CSF pressure factors at the time of CSF examination are associated with subsequent development of PDPH.

## Materials and Methods

### Participants

This study was conducted prospectively from October 2014 through June 2018. We enrolled participants who were scheduled to undergo CSF studies to detect meningitis.

Before CSF study, all participants were evaluated by brain CT, basic laboratory tests, clinical history, and neurologic examinations. We excluded participants with a history of neurologic disease (including primary and secondary headache disorders and postural headache), connective tissue disorders, previous dural puncture, or abnormal structural lesions on neuroimaging studies. Participants who underwent traumatic tap and failed dural puncture were also excluded. After confirmatory CSF analysis, we enrolled participants with aseptic meningitis or normal CSF findings.

### Study Design

To evaluate the relationship between pressure factors and PDPH, we performed dural puncture while controlling for the following factors related to PDPH. (1) Needle shape: Quincke needle, (2) needle size: 20–23 gauge, (3) bevel orientation: parallel to the long axis of the spine, (4) needle insertion location and angle: L4-5 interspace with 10-degree angle toward the umbilicus, (5) CSF drainage volume: 15 ml, (6) dural puncture position: side-lying position, (7) resting duration and hydration after dural puncture: 4 h and 80 ml/h with normal saline. Skilled neurologists performed all dural puncture procedures.

Once the CSF flow was established, a stopcock valve and manometer were attached to the spinal needle. To avoid false rises and drops of CSF pressure, we asked participants to relax and straighten their legs slowly. CSF OP was checked at the peak level of the manometer that showed slight up-down changes with respiration. Then, we opened the valve and collected CSF into a test tube. After CSF drainage was complete, we opened the manometer valve and measured CP in the same way.

### Diagnosis of PDPH

According to ICHD-3, the diagnostic criteria of PDPH include a headache that occurs within 5 days after dural puncture as well as criteria for identifying headaches attributed to low CSF pressure. Some previous studies reported that PDPH could develop between 5 and 14 days after dural puncture (7). Therefore, we confirmed the occurrence of PDPH on the fifth and 14th days after dural puncture. We defined PDPH as newly developed orthostatic headache after dural puncture with clearly different characteristics compared to baseline headache features. Orthostatic headache is defined below; when the participant is in the supine position, the headache improves more than 50% or to VAS score <3. When PDPH was suspected, we carefully observed the consistency of headache character for 2 days.

### Ethical Approval

This study was approved by the local ethics committee of Ujeongbu St. Mary's Hospital, Ujeongbu, Republic of Korea (approval no. UC17OESI0075). Written informed consent was obtained from all participants. For participants who were 18 years old or younger, we obtained written informed consent from their parents.

### Data Analysis

When the lumbar puncture procedure ended, the operator checked and described the pressure (OP, CP), procedure (needle size, trial number, drained CSF volume) and demographic factors. The craniospinal elastance (ECS) was calculated using OP, CP, and removed CSF volume (13).

$$\begin{array}{l} ECS=\frac{\Delta P}{\Delta V}=\frac{\left(OP-CP\right)}{drained\;volume} \end{array}$$

Pressure-volume index (PVI) is the CSF volume required to raise CSF pressure by a factor of 10.

$$\begin{array}{l} PVI=\frac{\Delta V}{\left(\log_{10}\frac{OP}{CP}\right)}=\frac{15}{\left(\log_{10}\frac{OP}{CP}\right)} \end{array}$$

The data described in the mean ± standard deviation. All statistical analyses were performed to test relationships between PDPH and controls using R (version 3.5.1). The Mann-Whitney *U*-test was used for numerical variables. Categorical variables were analyzed by the chi-square test with Fisher's correction when appropriate. According to diagnosis, statistical analysis performed between PDPH and non-PDPH in each aseptic meningitis and normal CSF group. Spearman correlation analysis was used to evaluate the relationships between factors that may be related to PDPH. Binary logistic regression analysis was performed to assess significant related factors for PDPH. All tests were two-tailed with a significance level of *p* < 0.05.

## Results

### Demographics

**Figure 1:**
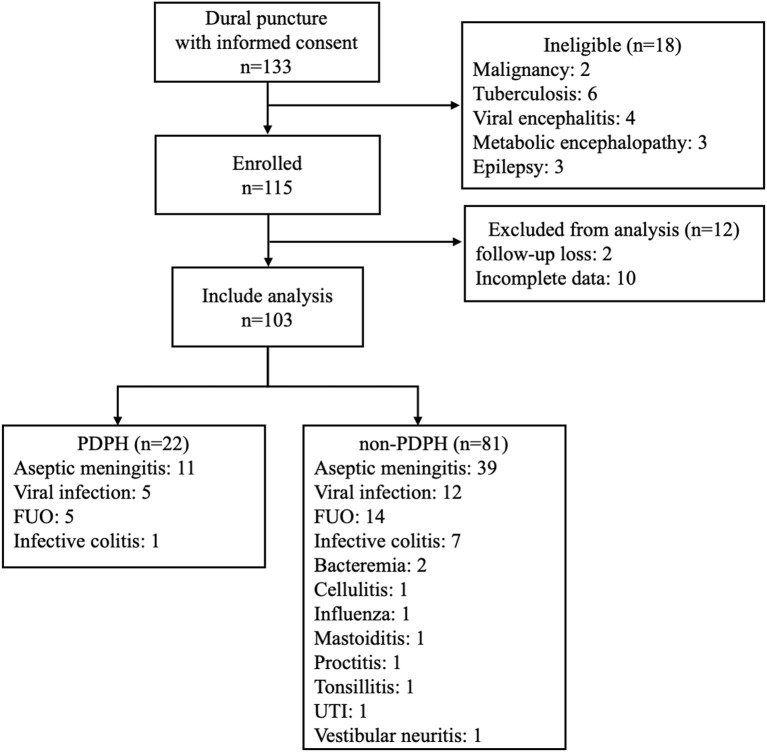
Flow chart of participants included in this study.

For this study, we enrolled 133 participants who visited our center with diagnoses of meningitis. We excluded 30 participants who did not match inclusion criteria, had incomplete data, or were lost to follow-up. Finally, 103 participants (53 male; age: 33.3 ± 13.5 years) were included in the analysis (Figure 1). CSF study confirmed aseptic meningitis in 50 participants (48.5%) and all others (53 participants, 51.5%) had normal CSF. Final diagnoses are described in Figure 1. PDPH developed in 22 participants (21.4%) at 1.91 ± 1.41 days after lumbar puncture. We treated 17 of 22 (77.27%) with autologous blood patches, while five (22.73%) spontaneously improved with bed rest.

### CSF Pressure Factor Analysis

**Figure 2:**
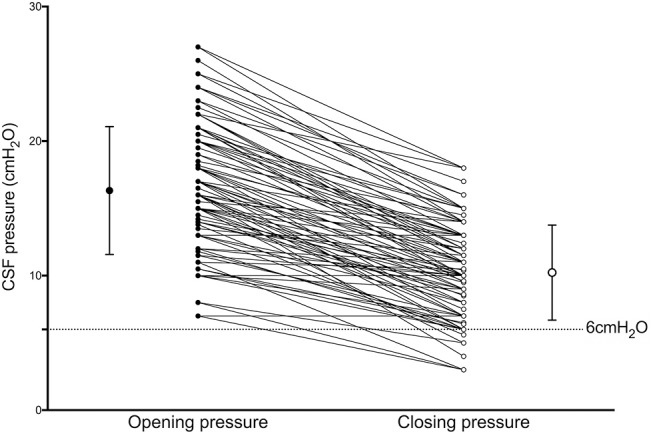
Results of Wilcoxon signed-rank tests between Opening (OP) and Closing (CP) pressure CP is significantly lower than OP (*p* < 0.001). Bars with whiskers indicate mean with standard deviation of OP (closed dot; 16.3 ± 4.7 cmH_2_O) and CP (open dot; 10.2 ± 3.5 cmH_2_O).

CSF OP was 16.3 ± 4.7 cmH_2_O, and CP was 10.2 ± 3.5 cmH_2_O. After draining CSF, 100 participants (97.1%) exhibited decreased CSF pressure with decreases of pressure (OP-CP) of 6.1 ± 3.1 cmH_2_O. CSF CP was lower than 6 mmH_2_O in 16 participants (15.5%) (Figure 2).

CSF OP showed a significant relationship with weight (*r* = 0.270, *p* = 0.006), and CP was also significantly correlated with weight (*r* = 0.347, *p* < 0.001) and height (*r* = 0.199, *p* = 0.047). ΔOP-CP showed a negative relationship with puncture trial number (*r* = −0.26, *p* = 0.024) and PVI was significantly correlated with weight (*r* = −0.020, *p* = 0.046) (Table 1).

**Table 1:** Correlations between CSF pressure factors and demographic characteristics.

	**Age**	**Sex (male)**	**Height**	**Weight**	**BMI**	**Trial**	**Needle**	**Volume**
OP	−0.175	0.170	0.181	0.270^**^	0.172	−0.145	−0.047	0.157
CP	−0.118	0.211^*^	0.199^*^	0.347^***^	0.19	0.039	0.063	0.108
OP-CP	−0.134	0.013	−0.036	0.028	0.041	−0.226^*^	−0.122	0.12
ECS	−0.136	−0.050	−0.036	−0.022	−0.05	−0.182	−0.089	−0.238
PVI	0.034	0.144	0.133	0.200^*^	0.173	0.145	0.079	0.319

Spearman's correlation coefficients are shown (*) indicates significant values

* *P < 0.05*;

** *P < 0.01*;

*** *P < 0.001*.

### CSF Pressure Factors and PDPH

All participants were divided into a PDPH group (*n* = 22) and a non-PDPH group (*n* = 81). CSF pressure factors include OP, CP, OP-CP, ECS, and PVI. None of these factors differed between groups (Table 2). Demographic characteristics and other CSF factors did not differ between the two groups. In subgroup analysis after dividing into two groups according to presence of viral meningitis, CSF pressure and procedural factors did not show the difference (Table 3). In bivariate logistic regression analysis, CSF pressure factors were not related to PDPH development (Table 4).

**Table 2:** Demographics, CSF variables, and procedural factors in PDPH and non-PDPH groups.

	**Total** **(*N* = 103)**	**PDPH** **(*N* = 22)**	**non-PDPH** **(*N* = 81)**	***p*-value**
**DEMOGRAPHICS**				
Age (years)	33.3 ± 13.5	29.5 ± 10.2	34.3 ± 14.1	0.133
Sex (male)	50 (48.5%)	10 (45.5%)	40 (49.4%)	0.931
Height (cm)	166.5 ± 8.8	167.1 ± 9.5	166.3 ± 8.6	0.710
Weight (kg)	64.8 ± 14.0	62.8 ± 12.8	65.3 ± 14.3	0.467
BMI	27.0 ± 7.5	24.6 ±7.2	27.6 ±7.5	0.099
**CSF ANALYSIS**				
Presence of CSF pleocytosis	50 (48.5%)	11 (50%)	39 (48.1%)	1.000
WBC count (/mm^3^)	15.1 ± 14.2	17.6 ± 16.1	14.4 ± 13.7	0.359
Protein (mg/dL)	61.6 ± 49.7	53.9 ± 46.3	63.6 ± 50.7	0.418
Glucose (mg/dL)	61.7 ± 16.2	57.8 ± 13.3	62.7 ± 16.8	0.212
**CSF PRESSURE FACTORS**				
Opening pressure (cmH_2_O)	16.3 ± 4.7	15.1 ± 4.0	16.7 ± 4.9	0.16
Closing pressure (cmH_2_O)	10.2 ± 3.5	9.6 ± 3.5	10.4 ± 3.5	0.363
OP-CP (cmH_2_O)	6.1 ± 3.1	5.4 ± 2.8	6.3 ± 3.2	0.263
Craniospinal elastance (ECS; ΔP/mL)	0.4 ± 0.2	0.4 ± 0.2	0.4 ± 0.2	0.326
Pressure volume index (PVI)	99.8 ± 89.5	107.2 ± 69.6	97.7 ± 94.7	0.659
proportion of participants with CP < 6 cmH_2_O	16 (15.5%)	5 (22.7%)	11 (13.6%)	0.325
**PROCEDURAL FACTORS**				
Trial number	2.3 ± 2.3	2.0 ± 1.2	2.4 ± 2.5	0.203
Needle size (gauge)	21.3 ± 0.8	21.3 ± 0.8	21.4 ± 0.8	0.782
Removed volume (ml)	15.5 ± 3.3	15.6 ± 3.5	15.4 ± 3.3	0.856

*P-values were determined by Mann Whitney-U-tests for numerical variables. Categorical variables were analyzed by chi-square tests with Fisher's correction when appropriate*.

**Table 3:** Subgroup analysis according to diagnosis.

	**Aseptic meningitis**			**Normal CSF**		
	**PDPH** **(*N* = 11)**	**non-PDPH** **(*N* = 39)**	***p*-value**	**PDPH** **(*N* = 11)**	**Non-PDPH** **(*N* = 42)**	***p*-value**
**CSF PRESSURE FACTORS**						
Opening pressure (cmH_2_O)	16.0 ± 3.0	17.3 ± 4.7	0.395	14.1 ± 4.7	16.1 ± 5.1	0.250
Closing pressure (cmH_2_O)	9.6 ± 3.7	10.3 ± 3.6	0.589	9.6 ± 3.5	10.5 ± 3.5	0.467
OP-CP (cmH_2_O)	6.3 ± 3.0	7.0 ± 2.6	0.502	4.5 ± 2.3	5.6 ± 3.5	0.334
Craniospinal elastance (ΔP/mL)	0.4 ± 0.2	0.5 ± 0.2	0.542	0.3 ± 0.2	0.4 ± 0.3	0.415
Pressure volume index (PVI)	97.3 ± 66.2	79.6 ± 41.2	0.417	117.2 ± 74.7	115.7 ± 125.6	0.970
proportion of CP < 6 cmH_2_O	3 (27.3%)	6 (15.4%)	0.392	2 (18.2%)	5 (11.9%)	0.626
**PROCEDURAL FACTORS**						
Trial number	2.0 ± 1.2	2.3 ± 2.3	0.551	1.9 ± 1.2	2.5 ± 2.7	0.254
Needle size (gauge)	21.3 ± 0.8	21.3 ± 0.7	0.857	21.5 ± 0.3	21.3 ± 0.6	0.421
Removed volume (ml)	15.9 ± 3.6	15.8 ± 3.2	0.920	15.3 ± 3.6	15.1 ± 3.4	0.895

*P-values were determined by Mann Whitney-U-tests for numerical variables and Fisher's exact tests for categorical variables*.

**Table 4:** Logistic regression analysis of factors associated with development of PDPH.

**Variables**	**B**	**SE**	***p*-value**	**Odds ratio (95% CI)**
**DEMOGRAPHICS**				
Age	−0.033	0.025	0.189	0.968 (0.918; 1.014)
Sex	−0.350	0.917	0.703	0.705 (0.110; 4.140)
Height	0.015	0.056	0.783	1.016 (0.909; 1.137)
Weight	0.030	0.033	0.364	1.031 (0.963; 1.099)
BMI	−0.080	0.051	0.119	0.923 (0.827; 1.014)
**CSF ANALYSIS**				
WBC count	−0.001	0.002	0.571	0.999 (0.995; 1.002)
Protein	−0.008	0.007	0.253	0.992 (0.976; 1.005)
Glucose	−0.041	0.032	0.196	0.960 (0.893; 1.006)
**CSF PRESSURE FACTORS**				
OP	−0.318	0.316	0.315	0.728 (0.398; 1.524)
CP	0.178	0.328	0.586	1.195 (0.551; 2.219)
OP-CP	−0.146	0.089	0.103	0.865 (0.720; 1.025)
ECS	−1.807	1.257	0.151	0.164 (0.012; 1.723)
PVI	0.006	0.007	0.405	1.006 (0.991; 1.021)
**PROCEDURAL FACTORS**				
Trial number	−0.207	0.176	0.239	0.813 (0.538; 1.101)
Needle size	0.222	0.369	0.548	1.249 (0.623; 2.696)
Removed volume	0.138	0.147	0.348	1.148 (0.851; 1.545)

## Discussion

In this study, we detected three significant outcomes. (1) After CSF drainage, CSF CP significantly decreased compared to OP. (2) Most CSF pressure factors were correlated with body weight. (3) CSF pressure factors are not related to PDPH development.

Because CSF is confined to a restricted space, it is predictable that the CSF pressure changes after CSF drainage (12). CP has been studied mainly in IIH, with varying results (9). A previous study reported that achieving a decrease of 1 cmH_2_O of pressure requires removing 0.91 ml of CSF (9). However, the pressure-volume relationship was not observed at pressures >15 cmH_2_O. Another recent study reported that when OP was below 20 cmH_2_O, pressure decreased by 0.52 cmH_2_O per 1 ml of drainage (13). Structural changes in the elasticity of the CSF lining were related to long-lasting intracranial pressure increments, and this relationship should be considered in such cases. There is no established reference range for CSF CP because it is affected by the volume removed (13). In a previous study, CSF pressure decreased after dural puncture, similar to our findings (6). The strength of present study is that we properly adjusted CSF drainage volume and influencing factors. Our results indicate that an average decrease of 0.4 ± 0.2 mmH_2_O occurs per 1 ml of CSF drained.

Clinically, obesity is related to IIH and weight gain increases the likelihood of developing IIH (16, 17). Women with IIH who lost weight had significantly reduced intracranial pressure and improved clinical symptoms (18). The mechanism underlying the relationship between body weight and intracranial pressure is still unclear, and there is a suggestion that increased intrathoracic and venous pressure due to abdominal mass affects the intracranial pressure by causing it to increase (19). A previous study reported BMI had a small but insignificant influence on CSF pressure in adults (20). Another study reported that CSF pressure increases by 3 cmH_2_O for every 10 units of BMI in children (21). The results of the present study suggest that body weight, more than overall obesity as assessed by BMI, affects CSF factors including OP, CP, and PVI.

Younger age, female sex low BMI, history of PDPH, and chronic headache are known as risk factors for the development of PDPH (6, 7). Previous studies of CSF CP and PDPH have been inconclusive (6). In our study, CSF CP was not associated with the development of PDPH. In 16 participants, CP was lower than 6 cmH_2_O, which is the diagnostic cutoff for PDPH, but there was no difference between PDPH and non-PDPH group (22.7% vs. 13.6%, *p* = 0.325). We may make several observations about these results. First, CSF pressure evaluations are not performed at the time of PDPH diagnosis, so restorative changes may occur in CSF pressure over time. Second, when PDPH occurs in a patient without a concomitant significant decrease of CP, we should consider the possibility of additional pressure drop due to persistent CSF leakage at the initial dural puncture point. In SIH patients, various imaging studies may be used to detect CSF leaks and focal CSF collection (22–24). CSF pressure is significantly lower in PDPH with definite leakage than in cases without leakage (15). Further research may be needed to assess the significance of CSF pressure at the time of PDPH and to analyze its relationship with CP at the time of initial dural puncture.

We found that decreases in CSF pressure and craniospinal elastance did not affect PDPH development. CSF OP-CP may roughly reflect the CSF reservoir. Large decreases in pressure indicate small reservoirs (12). In patients with intracranial hypertension, ECS is decreased, and PVI is increased (22). PVI significantly increases in cases of spontaneous intracranial hypotension (SIH) with definite CSF leaks compared to those without CSF leaks (15). Headaches related to CSF leaks are related to decreases of ECS and increases of PVI (22). We hypothesize that these factors may not be directly related to PDPH, but that more complex mechanisms such as vasodilation, pain sensitization, and neuroinflammation are involved in the development of PDPH (7, 19, 25).

The results of the present study indicate that there is no significant relationship between CSF-related procedures and PDPH. These results are inconsistent with those of previous studies. In most studies, patient posture, operator experience, needle shape, and needle size have been associated with PDPH development (7, 26, 27). The results of the present study require careful interpretation because we adjusted for these factors, which remained constant. The removed CSF volume can non-linearly influence the slope of ECS and PVI change during the drainage time course (13, 15). The previous study investigated the pressure-volume changes by using continuous ICP monitoring, but our study did not include monitoring procedure during CSF drainage (15). Because of the limitation of present study design, there is still the possibility that the difference in PCI and ECS depending on presence of PDPH may not be clarified. Further Investigation may need to reveal the relation between pressure-volume change and CSF removal by using CSF pressure monitoring could be helpful.

This study was conducted in a single institution, and the small number of participants included is a limitation of our study. We included patients with aseptic meningitis, so our results have limitations regarding generalization to other patients for PDPH interpretation. We did not assess intraventricular pressure or establish the presence of definite CSF leaks.

## Conclusion

Factors related to CSF pressure might not be related to the development of PDPH.

## Data Availability

All datasets generated for this study are included in the manuscript and/or the supplementary files.

## Ethics Statement

This study was approved by the local ethics committee of Ujeongbu St. Mary's Hospital, Ujeongbu, Republic of Korea (approval no. UC17OESI0075). Written informed consent was obtained from all participants. For participants who were 18 years old or younger, we obtained written informed consent from their parents.

## Author Contributions

SK, HS, SL, DR, TK, and JP conceived and designed the present study. HS, SL, and DR performed dural puncture procedures and acquired data. SK, TK, and JP analyzed and interpreted the data. SK and JP drafted the manuscript and made final decisions regarding the version to be published. JP agreed to be accountable for all aspects of the work and to ensure that questions related to the accuracy or integrity of any part of the work would be appropriately investigated and resolved.

### Conflict of Interest Statement

The authors declare that the research was conducted in the absence of any commercial or financial relationships that could be construed as a potential conflict of interest.
